# Hybrid contact force ablation: best of both worlds?

**DOI:** 10.1007/s12471-015-0723-4

**Published:** 2015-07-17

**Authors:** Y.J. Taverne, D. Merkus

**Affiliations:** 1 Department of Cardothoracic Surgery, Thoraxcenter Erasmus MC, Rotterdam, The Netherlands; 2 Cardiac Morphology, Department of Anatomy (ERCATHAN), Thoraxcenter Erasmus MC, Rotterdam, The Netherlands; 3 Division of Experimental Cardiology, Department of Cardiology, Thoraxcenter Erasmus MC, PO Box 2040, 3000 CA Rotterdam, The Netherlands

Atrial fibrillation (AF) is the most common clinically significant arrhythmia and is associated with increased mortality and morbidity, more specifically stroke and heart failure [1, 2]. The incidence and prevalence of AF is rising with increments of age, and, within an ageing population, an escalation of disease burden is to be expected [2]. During the past decade, major advances have been made in both surgical techniques for ablation of AF, using either standard or minimally invasive techniques, and catheter ablation of AF [3].

Over 15 years ago, foci of ectopic beats initiating at the pulmonary veins (PV) were recognised as a trigger for AF, thereby forming the basis for developing catheter ablation [4]. Ever since, the creation of electrical insulation lesions around the pulmonary veins has been the hallmark of catheter ablation. However, event-free survival rates differ based on patient characteristics and type of AF. The estimate of efficacy of a single-ablation procedure on an ‘optimal’ candidate with paroxysmal AF is approximately 60–80 %. Conversely, when performed in patients with persistent AF, the success rate drops to 50–70 % [5]. Also, anatomical variation of the pulmonary vein ostia plays a major role, as the goal in eliminating AF signals is to produce a lesion that spans the full thickness of the tissue. The Achilles’ heel of the technique is that it was soon recognised that early PV reconnection may occur after ablation [6].

Yokoyama’s group [7] was the first to describe an important link between the tissue contact force (CF) of the catheter and the size of the lesion, leading to the creation of more steerable sheaths and, finally, force-sensing catheters. This recent advance in catheter-based radiofrequency ablation allows measurement of electrode-tissue interaction during the ablation procedure, thereby facilitating optimisation of the CF required for optimal ablation [8], ensuring that energy is adequately transmitted to the tissue, while minimising the risks associated with very high contact forces, such as chances of thrombus formation, steam pop formation and cardiac perforation. Use of this CF catheter in the SMART-AF trial, a prospective, multicentre trial, improved outcome in that the success rate, defined as 12 months’ freedom from symptomatic atrial arrhythmias, was 81 vs. 66 % in a historical control study [9].

Another approach is to perform stand-alone AF surgery, which should be considered, as presented by the Expert Consensus Statement, for symptomatic AF patients who prefer a surgical approach or when one or more attempts at catheter ablation have failed, or for patients who are not candidates for catheter ablation. Although this approach achieves higher arrhythmia-free success rates after a single procedure, lesions are not always transmural [10]. Especially the isthmus lines of both the mitral and tricuspid valve are difficult to ablate from an epicardial aspect [11].

Recent collaboration between electrophysiologists and cardiac surgeons introduced the best of both worlds, thereby creating a convergent ablation which seems to be an attractive alternative to overcome limitations inherent to each approach [12].

In the present issue of the *Netherlands Heart Journal*, Kumar et al. [13] are the first to implement the CF catheter in such a hybrid approach. They showed that the CF catheter also improved outcome when used in a hybrid approach. After a median follow-up time of approximately 12 months, 33 out of 38 patients (87 %) remained in sinus rhythm without requiring antiarrhythmic drugs compared with 21 out of 30 patients (70 %) in the control group. Interestingly, all patients with recurrent arrhythmias retrospectively had a catheter-based touch-up in which CF was below 10 g, and thereby significantly below the optimal CF of 18 g, suggesting that CF had been suboptimal during the procedure. Importantly, the use of the CF catheter was associated with a shorter procedure time, and did not increase the number of serious adverse events [13].

Nonetheless, given the small number of patients and the single-centre nature of the study by Kumar et al., randomised multicentre clinical trials by experienced investigators are required, not only to determine the true usefulness of the CF catheters in a hybrid approach, but also to analyse whether a (staged) hybrid approach improves long-term event-free survival.

Importantly, in the future, further technological advances may allow more accurate preoperative localisation of AF triggers, which may further shorten procedure times and may lead to tailored treatment and improved outcome of ablation of AF [14]. Nevertheless, it should be noted that despite all the technological advances and better-quality treatment strategies that have led to improved outcomes of AF over the past decades, the pathophysiological mechanisms that underlie this frequently occurring phenomenon are still incompletely understood. It is well known that AF begets AF and in-depth analyses of this pathway may possibly provide more insight into AF pathophysiology. With the predicted growing number of patients with AF as a result of an ageing population and increasing comorbidities in the general population, a better understanding of these underlying mechanisms is imperative to reduce the number of AF patients and the costs associated with diagnosis and treatment of these patients to the healthcare system.

## Funding

None.

## Conflict of interest

None declared.
